# α-Tubulin Regulates the Fate of Germline Stem Cells in *Drosophila* Testis

**DOI:** 10.1038/s41598-021-90116-7

**Published:** 2021-05-20

**Authors:** Xiaoqian Tao, Yunqiao Dou, Guangyu Huang, Mingzhong Sun, Shan Lu, Dongsheng Chen

**Affiliations:** 1 Anhui Provincial Key Laboratory of the Conservation and Exploitation of Biological Resources, College of Life Sciences, Anhui Normal University, Wuhu, 241000 China; 2 Anhui Provincial Key Laboratory of Molecular Enzymology and Mechanism of Major Diseases, College of Life Sciences, Anhui Normal University, Wuhu, 241000 China; 3 College of Life Sciences, The Institute of Bioinformatics, Anhui Normal University, Wuhu, 241000 China

**Keywords:** Stem cells, Self-renewal

## Abstract

The *Drosophila* testis provides an exemplary model for analyzing the extrinsic and intrinsic factors that regulate the fate of stem cell in vivo. Using this model, we show that the *Drosophila αTub67C* gene (full name *αTubulin at 67C*), which encodes α4-Tubulin (a type of α-Tubulin), plays a new role in controlling the fate of male germline stem cells (GSC). In this study, we have found that *Drosophila* α4-Tubulin is required intrinsically and extrinsically for GSCs maintenance. Results from green fluorescent protein (GFP)-transgene reporter assays show that the gene *αTub67C* is not required for Dpp/Gbb signaling silencing of *bam* expression, suggesting that *αTub67C* functions downstream of or parallel to *bam*, and is independent of Gbb/Dpp-*bam* signaling pathway. Furthermore, overexpression of *αTub67C* fails to obviously increase the number of GSC/Gonialblast (GB). Given that the *α-tubulin* genes are evolutionarily conserved from yeast to human, which triggers us to study the more roles of the gene *α-tubulin* in other animals in the future.

## Introduction

Tissue maintenance and regeneration rely on adult stem cells (ASCs), which are characterized by their ability to constantly reproducing themselves (self-renewal). At the same time, ASCs are also capable of producing new differentiated cells (differentiation) to replenish many tissues such as skin, gut, gonad, blood and muscle^1^. ASCs play an essential role in tissue homeostasis by maintaining a balance between self-renewal and differentiation. Numerous studies from diverse systems have shown that this balance is controlled by both intrinsic regulators in ASCs and extrinsic signals from the microenvironment (called “niche”) surrounding ASCs^2^. Germline stem cells (GSCs) in the *Drosophila* testis provide an excellent model for studying of the mechanisms of ASCs fate determination in vivo ^3^.

**Figure 1:**
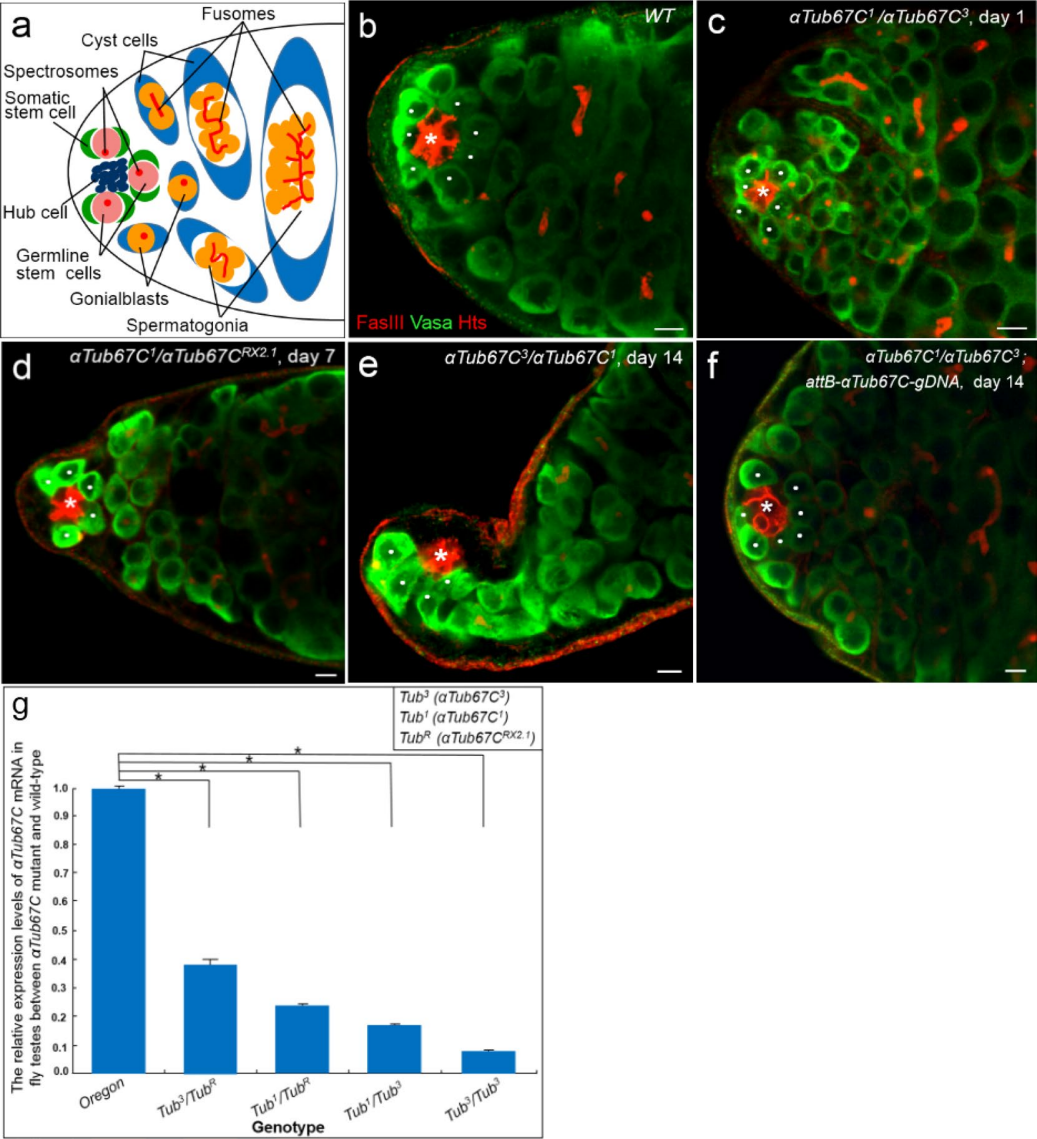
*αTub67C* is required for maintaining GSCs in *Drosophila* testis. **(a)** A schematic diagram of an adult testis. GSCs (pink), Hub cells (dark blue), Somatic stem cells (green), Gonialblasts (yellow), cyst cells (blue), and fusomes (red). (**b)** Testis from the wild-type (*WT*) fly. **(c–e)** *αTub67C* mutant testes at different ages showed the GSC loss phenotypes. **(f)** The transgene P{*attB-αTub67C-gDNA*} rescued the *αTub67C* mutant testis to normal. (**g)** Quantitative PCR analyses of *αTub67C* mRNA levels in testes between wild-type and *αTub67C* mutants. Testes stained with anti-Fas III antibody to label the hubs (red, indicated by asterisks), anti-Hts antibody to label the fusomes (red), and anti-Vasa antibody to label germ cells (green) (**b**–**f**). GSCs were highlighted by white dots. Testes with 7 GSCs (**b** and** f**), 6 GSCs (**c**), 5 GSCs (**d**) and 4 GSCs (**e**) are shown. *Oregon-R* was used as the wild-type flies. Scale bars: 5 μm. *p < 0.001.

Adult male *Drosophila* has a pair of testes, each of which is a long blind-ended tube coiling around a seminal vesicle. A cluster of 10–15 non-mitotic somatic cells called the hub resides at the blind apical end of each adult testis (Fig. 1a). GSCs undergo asymmetric divisions, generating one of the daughter cells that remains adjacent to the hub (as the niche for GSCs) and remains a stem cell, and the other one, called the gonialblast (GB), which is displaced away from the hub and initiates differentiation. GSC can be marked by a dot-like spectrosome which is positioned at the anterior in the cells, while the spectrosome in GB usually lose the anterior localization (Fig. 1a). The GB progresses through four rounds of mitotic divisions with incomplete cytokinesis to form a cluster of 16-cells spermatogonia interconnected by a branched fusome (Fig. 1a). Actually, the fusome and spectrosome are the same organelle that changes shape throughout differentiation. Spermatogonia differentiate into spermatocytes, which undergo meiosis and finally form sperms. Each GSC is enwrapped by two cyst stem cells (CySC). CySCs retain attached to the hub and differentiate into cyst cells, which encapsulate the gonialblast and its progeny during spermatogenesis. Both the hub cells and CySCs serve as the niche for GSCs, while CySCs only depend on the hub cells for niche signals^4,5^.

Previous studies have shown that several signaling pathways regulate the fate of GSCs in *Drosophila* testis. Bone morphogenetic protein (BMP) signaling is crucial for GSCs maintenance in *Drosophila* testis. Two BMP ligands, Decapentaplegic (Dpp) and Glass bottle boat (Gbb), are expressed in these two types of niche cells (the hub cells and CySCs) and activate signaling in GSCs^6,7^. GSCs self-renewal require BMP pathway activation to repress transcription of the differentiation factor *bag of marble* (*bam*)^6–8^. Loss of BMP in niche cells or lack of downstream BMP pathway components in GSCs leads to the loss of the GSCs phenotype^6–8^. Janus kinase-signal transducer and activator of transcription (JAK-STAT) signaling pathway is also required for the maintenance of both GSCs and CySCs^9,10^. Interestingly, the self-renewal of GSCs is not directly due to activation of JAK-STAT in GSCs, but due to JAK-STAT activation in CySCs, which results in the consequent increased expression of BMP ligands from CySCs^11–13^. Similarly, recent studies show that Hedgehog (Hh) signaling activity in CySCs also positively regulates BMP signaling activity in *Drosophila* testis to maintaining GSCs, and the loss of Hh signaling in CySCs leads to precocious differentiation of GSCs^14^. Therefore, it is reasonable to thought that BMP signaling is the primary pathway maintaining GSCs in *Drosophila* testis^13^. In addition, some intrinsic factors that regulate the testis GSCs fate in *Drosophila* have been identified, such as Nop60B, DBHD, Lola, Piwi, Gilgamesh and Maf-S^15–20^.

It is well known that the α- and β-Tubulins are conserved throughout the evolution of eukaryotes, and the heterodimers of α/β- Tubulin primarily constitute the structural subunits of microtubule (MT), which has several important functions (e.g. existing in eukaryotic cells as a type of cytoskeleton filaments to sustain the cell shape, forming some specialized structures including cilia, flagella and mitotic spindles)^21–23^. *Drosophila αTub67C* gene (full name *αTubulin at 67C*), which encodes α4-Tubulin (a type of α-Tubulin), has been involved in regulating multiple physiological processes, such as oocyte meiosis, neurogenesis, centromere positioning, centrosome segregation, lipid-droplet transport, spindle elongation and the formation of the sperm aster^24–29^. In the present paper, we use mutant *αTub67C* alleles to reveal a novel function of α4-Tubulin in maintaining the fate of germline stem cells in *Drosophila* testis.

## Results

### Deficiency of *αTub67C* influences GSCs maintenance in *Drosophila* testis

To identify genes that potentially control the GSC fate, we performed a screen of male lines mutagenized by radial ray in *Drosophila*. We isolated a line with a mutation in the third chromosome, *αTub67C*^3^ (X-ray-induced mutant allele)^24^, and found that some *αTub67C*^3^ homozygous mutant flies (20%, n > 100) exhibited shrunk testes at day 10 after eclosion (Fig. S1). To determine whether *αTub67C* affect the behavior of GSCs, we obtained two additional alleles, *αTub67C*^*RX2.1*^ (X-ray) and *αTub67C*^1^ (ethyl methanesulfonate-induced mutant allele)^24,25^. Then through genetic crosses, the testes of mutant flies collected at days 1, 7 and 14 after eclosion were stained with anti-Fas III, anti-Vasa and anti-Hts antibodies, and the number of GSCs was measured. Fas III is specifically expressed in hub cells (a cluster of somatic cells located to the tip of testis), whereas both Vasa and Hts are present in germ cells (Fig. 1b)^6,30^. Moreover, Hts is preferentially rich both in spherical spectrosomes and branched fusomes (two organelle-like structures made of cytoskeleton in germ cells) (Fig. 1a,b)^30^. In the wild-type (*Oregon R* flies was used as the wild-type control in this research) testis, 6–10 GSCs can be reliably recognized by at least three characteristics: anti-Vasa staining, containing a round spectrosome and directly attaching to the hub cells^30^. Additionally, a germline linage with sequentially differentiated spermatogonial cells (containing 2-, 4-,8- and 16-cells) marked by branched fusomes were also observed (Fig. 1a,b).

According to the method described previously^19^, we first quantified the GSC number in *αTub67C* heterozygous testes at three different ages (Table 1). It was similar to wild-type that *αTub67C*^*3*^ heterozygous males (*αTub67C*^*3*^*/* +) had a normal GSC number, which was counted as 7.8, 7.4 and 7.0 GSCs/testis at days 1, 7 and 14 after eclosion, respectively. The testes from the remaining two heterozygotes (*αTub67C*^*RX2.1*^*/* + and *αTub67C*^*1*^*/* +) contained an average of 7.6 and 7.5 GSCs/testis at day 1, respectively. Interesting, two weeks after being cultured at room temperature (RT), the testes from these two *αTub67C* heterozygotes contained an average of 6.7 and 5.8 GSCs/testis, respectively (Table 1). The data preliminarily indicate that deficit of one copy of gene *αTub67C* leads to a slight loss of GSCs.

**Table 1:** Phenotypic assay for *αTub67C* mutant flies. SD, standard deviation. n, Number of testes examined. ^***#***^*P* > 0.05; ^##^*P* < 0.05; ^***∗***^*P* < 0.01, unpaired t-test, compared with *Oregon-R* at day 14.

Genotype	The average number of GSCs in *Drosophila* testis at different ages (Mean ± SD)		
	Day 1	Day 7	Day 14
*Oregon-R*	8.1 ± 1.0 (n = 68)	7.8 ± 0.9 (n = 70)	7.4 ± 1.0 (n = 67)
*αTub67C*^*3*^*/* +	7.8 ± 0.9 (n = 65)	7.4 ± 0.9 (n = 68)	7.0 ± 1.3 (n = 62)^#^
*αTub67C*^*RX2.1*^*/* +	7.6 ± 1.1 (n = 58)	6.8 ± 1.0 (n = 65)	6.7 ± 1.1 (n = 65)^##^
*αTub67C*^*1*^*/* +	7.5 ± 0.9 (n = 66)	7.2 ± 0.9 (n = 69)	5.8 ± 1.9 (n = 71)^##^
*αTub67C*^*3*^*/αTub67C*^*3*^	6.1 ± 1.0 (n = 66)	5.2 ± 1.2 (n = 62)	4.2 ± 1.2 (n = 63)^∗^
*αTub67C*^*3*^*/αTub67C*^*RX2.1*^	6.2 ± 1.0 (n = 76)	5.5 ± 1.3 (n = 67)	4.9 ± 1.2 (n = 70)^∗^
*αTub67C*^*1*^*/αTub67C*^*RX2.1*^	6.0 ± 0.9 (n = 68)	5.2 ± 1.1 (n = 65)	4.5 ± 1.1 (n = 57)^∗^
*αTub67C*^*3*^*/αTub67C*^*1*^	6.0 ± 0.8 (n = 68)	5.0 ± 1.2 (n = 70)	3.9 ± 1.0 (n = 76)^∗^

We next quantified the number of GSCs in the testes of different *αTub67C* mutants at days 1, 7 and 14 post-eclosion. In the three time points, *αTub67C*^*3*^ homozygous testes carried an average of 6.1, 5.2 and 4.2 GSCs/testis respectively (Table 1), exhibiting a notable GSCs loss over the past 14 days. Similar results were observed in *αTub67C* trans-heterozygous mutants, *αTub67C*^*3*^*/αTub67C*^*RX2.1*^, *αTub67C*^*1*^*/αTub67C*^*RX2.1*^ and *αTub67C*^*3*^*/αTub67C*^*1*^. These three trans-heterozygous *αTub67C* mutants contained an average of 6.2, 6.0 and 6.0 GSCs/testis, respectively, at day 1 (Fig. 1c and Table 1). One week after being cultured at RT, these three *αTub67C* mutants had an average of 5.5, 5.2 and 5.0 GSCs/testis respectively (Fig. 1d and Table 1), whereas the wild-type contained a normal GSC number (7.8 GSCs/testis). At day 14, the average GSCs number was dramatically reduced to 4.9, 4.5 and 3.9 GSCs/testis respectively (Fig. 1e and Table 1). By contrast, the average number of GSCs from wild-type testes was sustained at normal level (7.4 GSCs/testis) (Table 1). These statistical data indicate that *αTub67C* is essential for maintaining GSCs in *Drosophila* testis.

To confirm a specific role of *αTub67C* in GSC maintenance, we performed a rescue assay by constructing a transgene of P{*attB*-*αTub67C-gDNA*}, in which a 7.3 kb genomic DNA fragment (containing 5.0 kb promoter, 2.0 kb exon/intron region and 0.3 kb 3’UTR fragment for *αTub67C*) was introduced into *attP*-*phiC31* fly hosts by *attB/attP*-element-mediated germline transformation^31^. We found that GSC loss phenotypes in three *αTub67C* allelic mutants were fully rescued by this transgene (Fig. 1f and Supplementary Table S1). Taken together, our results definitely suggest that *αTub67C* plays an essential role in GSCs maintenance.

To determine whether *αTub67C* mutation reduces the expression of *αTub67C* in fly testes, we performed real-time quantitative PCR (qPCR) assays to compare the mRNA level between the wild-type and mutant fly testis^32^. According to the previously described method^33^, we extracted total RNA from *Drosophila* testes, conducted reverse-transcription (RT) and performed qPCR experiments to measure the whole *αTub67C* mRNA level with the *rp49* gene as a reference. Compared with wild-type, the *αTub67C* mRNA expression level in *αTub67C* mutant testes (*αTub67C*^*3*^*/αTub67C*^*RX2.1*^, *αTub67C*^*1*^*/αTub67C*^*RX2.1*^, *αTub67C*^*3*^*/αTub67C*^*1*^ and *αTub67C*^*3*^*/αTub67C*^*3*^) was reduced significantly (Fig. 1g). These results strongly suggest that α4-Tubulin is reduced in *αTub67C* mutant testes, implying that the α4-Tubulin protein is responsible for the loss of GSCs phenotype in *αTub67C* mutant flies.

The self-renewal of GSCs critically depends on its adhesion to hub^1,4^. Since tubulin protein functions as a cytoskeleton filament, whether the cell adhesion between hub cell and GSC is affected in *αTub67C* mutation background. To explore whether the *αTub67C* mutant GSCs lose adhesion to the hub, we labeled germ cells (including GSCs) with anti-Vasa antibody and stained the testes with FITC-conjugated Phalloidin^19^. We observed that, just like the wild-type control (Fig. S2a), the GSCs were adhered tightly to hub cells both in *αTub67C*^*3*^*/αTub67C*^*1*^ (n > 90) and in *αTub67C*^*3*^*/αTub67C*^*3*^ mutant testes (n > 80) collected at day 14 post-eclosion (Fig. S2b,c). The data indicate that the gene *αTub67C* doesn’t regulate cell–cell (GSC and hub cell) adhesions in *Drosophila* testis, suggesting some other mechanisms maybe responsible for the GSCs loss phenotype.

The above results showed that the *αTub67C* mutant GSCs in fly testes were progressively lost with the time lapse. To explore whether loss of GSCs in *αTub67C* mutants was caused by its apoptosis-mediated cell death^19^, we examined the rate of apoptosis in *αTub67C* mutant GSCs by Terminal deoxynucleotidyl transferase-mediated dUTP Nick End Labelling (TUNEL) assays^34^. We found that there was no cell apoptosis in GSCs both from wild-type (*Oregon*) testes and from two *αTub67C* mutants (*αTub67C*^3^/*αTub67C*^1^ and *αTub67C*^3^/*αTub67C*^3^) at day 7 post-eclosion, and only found apoptosis-occuring in GBs/spermatagonia (Fig. S3a,b). We also determined the apoptosis rate of marked mutant GSC clones, according to the method described previously^19^. Similar results were observed in mutant GSC clones, there was no apoptosis-occurring in *αTub67C* mutant GSC clones (Fig. S3c,d). These results suggest that mutant GSCs may precociously differentiate into GBs.

### The gene *αTub67C* regulates the GSC fate both intrinsically and extrinsically

Previous studies have shown that GSC self-renewal is controlled by regulators that function inside the GSCs or in the niche cells, or both^9,35–37^. To further determine the role of *αTub67C* in GSC maintenance, we examined the expression profile of αTub67C in fly testes employing a newly constructed transgenic reporter, P{*αTub67cP-αTub67C-gfp*}, in which the *αTub67C-gfp* fusion coding sequence was placed under the control of a 5.0 kb *αTub67C* promoter. Thus, GFP expression can be used to represent that of *αTub67C*. By immunostaining testes with an anti-GFP antibody (Fig. S4), we observed that the αTub67C protein was ubiquitously expressed in all cell types including somatic cells (e.g. hub) and germline cells (e.g. GSCs and GBs) in transgenic fly testes (n > 80), suggesting that *αTub67C* functions in GSCs or the niche cells, or both. However, whether *αTub67C* works as an intrinsic or extrinsic modulator remains elusive.

**Figure 2:**
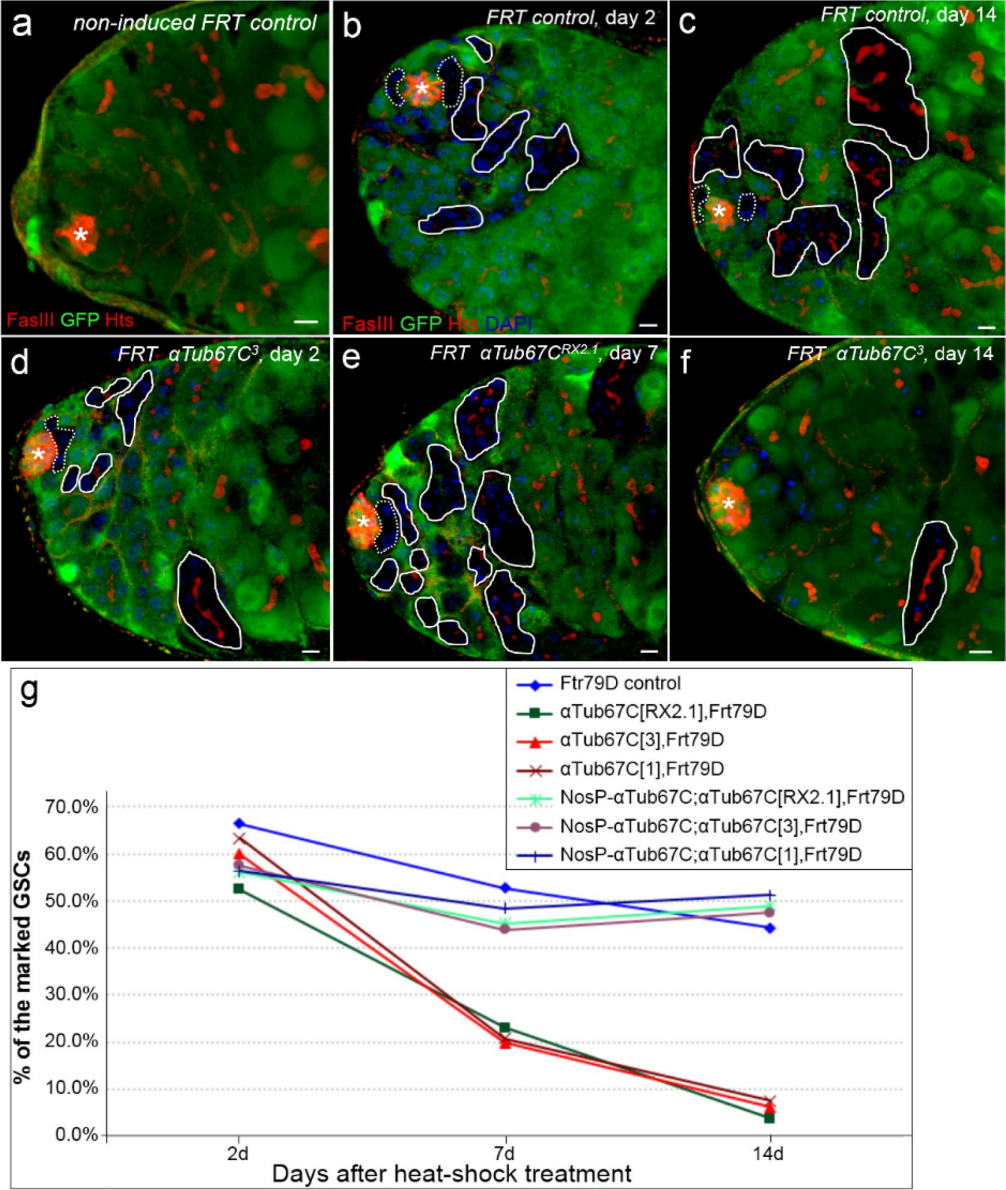
Intrinsic mutation of *αTub67C* leads to GSCs loss in *Drosophila* testis. (**a**) Testis from non-heat shock *FRT* control. Testes from *FRT* control (**b**,**c**) and *FRT αTub67C* flies (**d**–**f**) were collected at the indicated days after heat-shock treatment. All testes were stained with anti-Fas III antibody (red) to label the hub (**a** red cluster of cells), anti-Hts antibody (red) to visualize fusomes, and anti-GFP antibody (green) to show the *αTub67C* expression pattern. (**b**–**f**) Testes with DAPI dye (blue) staining to mark the nuclei. Hubs were noted by asterisks. GSCs clones (indicated by broken lines) and GB/spermatogonia clones (indicated by circles) were identified by lack of GFP expression. (**g**) Percentages of negatively GFP-marked GSC clones in *FRT* control and *αTub67C* mutant alleles at day 2, 7 and 14. Compared with *FRT* control, the percentages of marked GSCs (lack of GFP expression, GFP-) in *αTub67C* mutants were dramatically declined. Scale bars: 5 μm.

To address this issue, we used the FLP (flipase)-mediated *FRT* mitotic recombination technique to generated marked *αTub67C* mutant GSC clones^19,38^. The *αTub67C* mutant GSCs were GFP-negatively marked after several days of heat-shock treatments. We analyzed the loss rate of marked GSCs, according to the method described previously^19,36^. In this experiment, we generated the *αTub67C* mutant GSC clones with no GFP expression after 4-day-heat-shoch treatments. We counted and compared the number of GFP negatively-marked GSCs between the *FRT* control (*hs-flp/* + *; FRT79D/FRT79D*) and the *αTub67C* mutant GSC clones (*hs-flp/* + *; αTub67C, FRT79D/αTub67C, FRT79D*), at days 2, 7 and 14 after heat-shock treatments (AHT) (Fig. 2 and Supplementary Table S2). In the non-heat-shock *FRT* control, GFP was expressed ubiquitously in *Drosophila* testis (Fig. 2a). For *FRT* control, the initial rates of marked GSC clones was 66.4% (n = 118, the “n” means the total number of GSCs) at day 2 AHT, and the final 44.3% (n = 111) at day 14 AHT (Fig. 2b,c,g). The data suggested that only 33.3% of the marked GSCs were lost during the 12-day AHT period. By contrast, the rates of marked *αTub67C* mutant GSC clones (*FRT αTub67C*^*RX2.1*^, *FRT αTub67C*^*3*^ and *FRT αTub67C*^*1*^) declined rapidly from the initial 52.4% (n = 113), 59.9% (n = 112) and 63.3% (n = 120), respectively, at day 2 AHT, to the final 3.7% (n = 115), 6.1% (n = 123) and 7.5% (n = 113), respectively, at day 14 AHT (Fig. 2d–f,g). These results suggested that 92.9%, 89.8% and 88.2% of marked *αTub67C*^*RX2.1*^, *αTub67C*^*3*^ and *αTub67C*^*1*^ mutant GSCs were lost during the course of the experiment. These findings indicate that *αTub67C* plays an intrinsic role for GSCs maintenance.

We next performed a rescue assay to substantiate the above point, by supplementing α4-Tubulin function in *αTub67C* mutant GSCs clones using *αTub67C-*coding fragment. We constructed a transgenic line, P{*nosP-αTub67C*}, in which the wild type *αTub67C* coding sequence was driven by the promoter of the gene *nanos* that shares a high expression level in germ cells^19^. We found that, compared to *αTub67C* mutant clones, in *αTub67C-*expressing testes, the ratios of marked *αTub67C* GSCs clones (*nosP-αTub67C; FRT αTub67C *^*RX2.1*^*, nosP-αTub67C; FRT79D αTub67C*^*3*^*,* and *nosP-αTub67C; FRT αTub67C*^*1*^) decreased very weakly, from the initial 56.1% (n = 120), 57.6% (n = 114) and 56.3% (n = 110), respectively, at day 2 AHT, to the final 49.0% (n = 127), 47.5% (n = 117) and 50.2% (n = 121), respectively, at day 14 AHT (Fig. 2g). The data support the conclusion that *αTub67C* intrinsically plays a role in maintaining GSCs.

**Figure 3:**
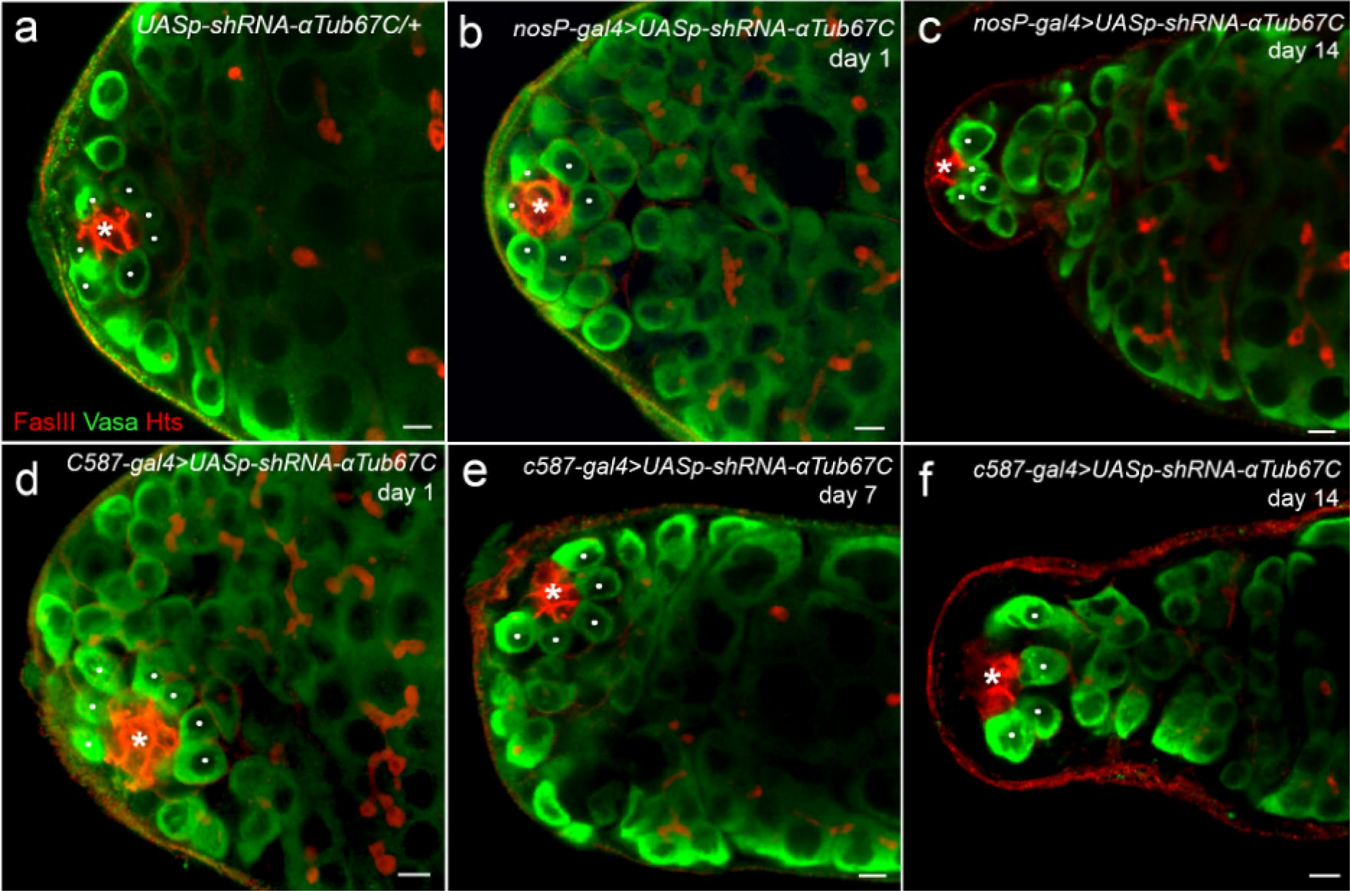
Intrinsic and extrinsic knockdown of *αTub67C* caused loss of GSCs in *Drosophila* testis. Testes stained with anti-Fas III antibody (red, indicated by asterisks), anti-Hts antibody (red), and anti-Vasa antibody (green). GSCs were noted by white dots. (**a**) The parental control testis with seven GSCs. The intrinsic *αTub67C*-knockdown (*nosP-gal4* > *UASp-shRNA-αTub67C*) flies (**b**,**c**) and the extrinsic *αTub67C*-knockdown (*c587-gal4* > *UASp-shRNA-αTub67C*) flies (**d**–**f**) collected at different days after eclosion. Testes containing 6 GSCs (**b**), 4 GSCs (**c**), 7 GSCs (**d**), 5 GSCs (**e**) and 4 GSCs (**f**). Scale bars: 5 μm.

To confirm the conclusion, we performed gene knockdown assay in fly testes employing the Gal4 > *UASp-shRNA* technique^37,39–41^. In this study, we specifically knocked down *αTub67C* in fly testes by combining P{*UASp-shRNA-αTub67C*} with P{*nosP-gal4*}. The short hairpin RNAs targeting *αTub67C* transcripts were produced by the germ cell-specific driver (*nosP-gal4*)^19^. Here, note that all of the tested flies were cultured at 29 ℃ to obtain a higher level of Gal4 activity, which can cause the increased phenotypic severity^42^. As shown in Table 2, we observed, in parental control testes (*UASp-shRNA-αTub67C/* +), the average GSC number was maintained at high level, counted as 8.0, 7.4 and 7.2 GSCs/testis at days 1, 7 and 14 post-eclosion (Fig. 3a), respectively. By contrast, in *αTub67C* intrinsic knockdown testes (*nosP-gal4* > *UASp-shRNA-αTub67C*), the average GSC number was dramatically decreased, counted as 7.7, 6.5 and 4.0 GSCs/testis at three ages post-eclosion, respectively (Fig. 3b,c and Table 2). The results support the point that *αTub67C* has an intrinsic role in GSCs maintenance.

**Table 2:** Phenotypic assay for the *αTub67C*-specific knockdown in *Drosophila* testis. All the examined flies were cultured at 29 ℃.SD, standard deviation. n, Number of testes examined. ^∗^*P* < 0.01, unpaired *t*-test, compared with parental control at day 14.

Genotype	The average number of GSCs in *Drosophila* testis at different ages (Mean ± SD)		
	Day 1	Day 7	Day 14
*UASp-shRNA-αTub67C/* +	8.0 ± 0.9 (n = 69)	7.4 ± 0.7 (n = 72)	7.2 ± 1.1 (n = 80)
*UASp-shRNA-αTub67C; nosP-gal4*	7.7 ± 1.1 (n = 73)	6.5 ± 0.9 (n = 79)	4.0 ± 0.8 (n = 73)^∗^
*UASp-shRNA-αTub67C; c587-gal4*	7.3 ± 1.1 (n = 82)	5.7 ± 0.9 (n = 77)	4.1 ± 1.0 (n = 79)^∗^

Whether *αTub67C* also plays an extrinsic role in maintaining GSCs? To address the hypothesis, we knocked down *αTub67C* in CySCs (one type of niche cells) by placing P{*UASp-shRNA-αTub67C*} under somatic driver (*c587-gal4*)^19^. Since it has been reported that C587-Gal4 is expressed in CySCs and early cyst cells but not in hub cells^6^, which triggers us to check whether *αTub67C* expresses in CySCs. By immunostaining the testes from transgenic flies of *αTub67cP-αTub67C-gfp* with anti-GFP and anti-Vasa antibodies, we observed that the αTub67C protein was expressed in CySCs as well as in hub cells. (Fig. S4a’’). Then we measured the average GSCs number in *αTub67C* extrinsic knockdown testes (*c587-gal4* > *UASp-shRNA-αTub67C*). Compared to parental control, strikingly, the average GSCs number from *αTub67C* extrinsic knockdown testes examined at three time points (day 1, 7 and 14 after eclosion) were decreased noticeably, measured as 7.3, 5.7 and 4.1 GSCs per testis, respectively (Fig. 3d–f and Table 2). The data suggest that *αTub67C* also plays an extrinsic role in niche cells for GSCs maintenance.

### *αTub67C* is not required for Dpp/Gbb signaling silencing of *bam*

**Figure 4:**
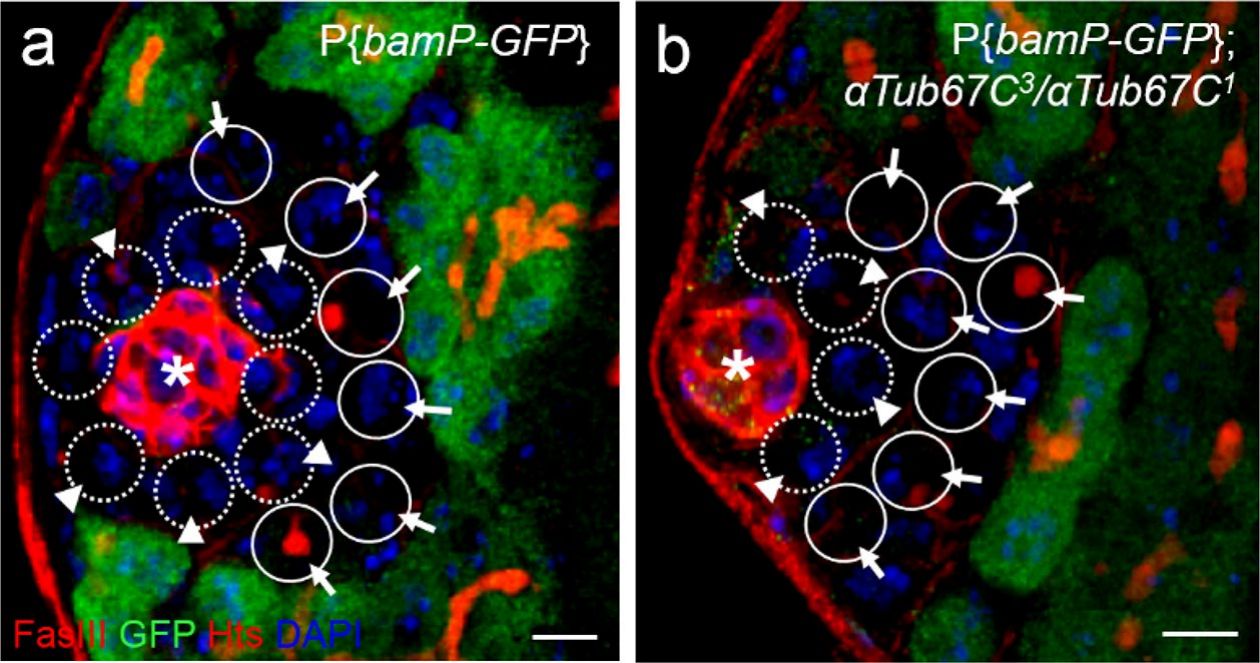
*αTub67C* fails to affect the expression patterns of *bam*. The testes were marked with Fas III antibody (red, hub with asterisk), Hts antibody (red, fusomes), GFP antibody (green) and dye DAPI (blue). Testes from *bamP-gfp* (**a**) and *bamP-gfp; αTub67C*^3^/*αTub67C*^1^ (**b**) male flies show negative GFP expression in either GSCs (indicated by arrowheads) or GBs (indicated by arrows). GSCs (highlighted by broken lines) can be recognized by their direct contact to hub (marked with asterisk) with DAPI staining (blue, cell nucleus) (Some GSCs can be observed anti-Hts staining). GBs (highlighted by circles) are far away from the hub but surround GSCs with DAPI staining (blue, cell nucleus) (Some GBs can be observed anti-Hts staining). Scale bars: 5 μm.

It has been reported that two Bmp members, Decapentaplegic (Dpp) and Glass bottle boat (Gbb), are co-expressed and function cooperatively to maintain GSCs in *Drosophila* testis by silencing of *bam* transcription^6^. To test whether *αTub67C* is engaged in Dpp/Gbb-dependent *bam* silencing, we analyzed the *bam* expression patterns in *αTub67C* mutant testes, by observing the GFP expression in GFP-transgene reporter, P{*bamP-GFP*}, in which a GFP coding sequence was driven by a *bam* promoter^43^. As shown in Fig. 4, the germ cells in testes from 7-day-old flies were marked with two antibodies (anti-GFP and anti-Hts) and 4’,6-diamidino-2-phenylindole (DAPI) staining. We found that the percentages of GSCs exhibiting a negative GFP pattern were 98.6% (n = 72 testes) in wild-type (*bamP-GFP*) and 98.8% (n = 83 testes) in *αTub67C* mutant flies (*αTub67C*^*3*^*/αTub67C*^*1*^), respectively (Fig. 4a,b). The data showed that there was no difference in *bam-GFP* expression pattern between wild-type and *αTub67C* mutant GSCs (*P* > *0.05*). Similarly, the ratios of GFP negatively-stained GBs between wild-type and *αTub67C* mutants were 98.7% (n = 68 testes) and 98.9% (n = 88 testes), respectively (Fig. 4a,b). Taken together, these results convincingly indicate that *αTub67C* is not required for Dpp/Gbb signaling silencing of *bam*.

### Ectopic overexpression of *αTub67C* fails to increase the number of GSC/GBs

**Figure 5:**
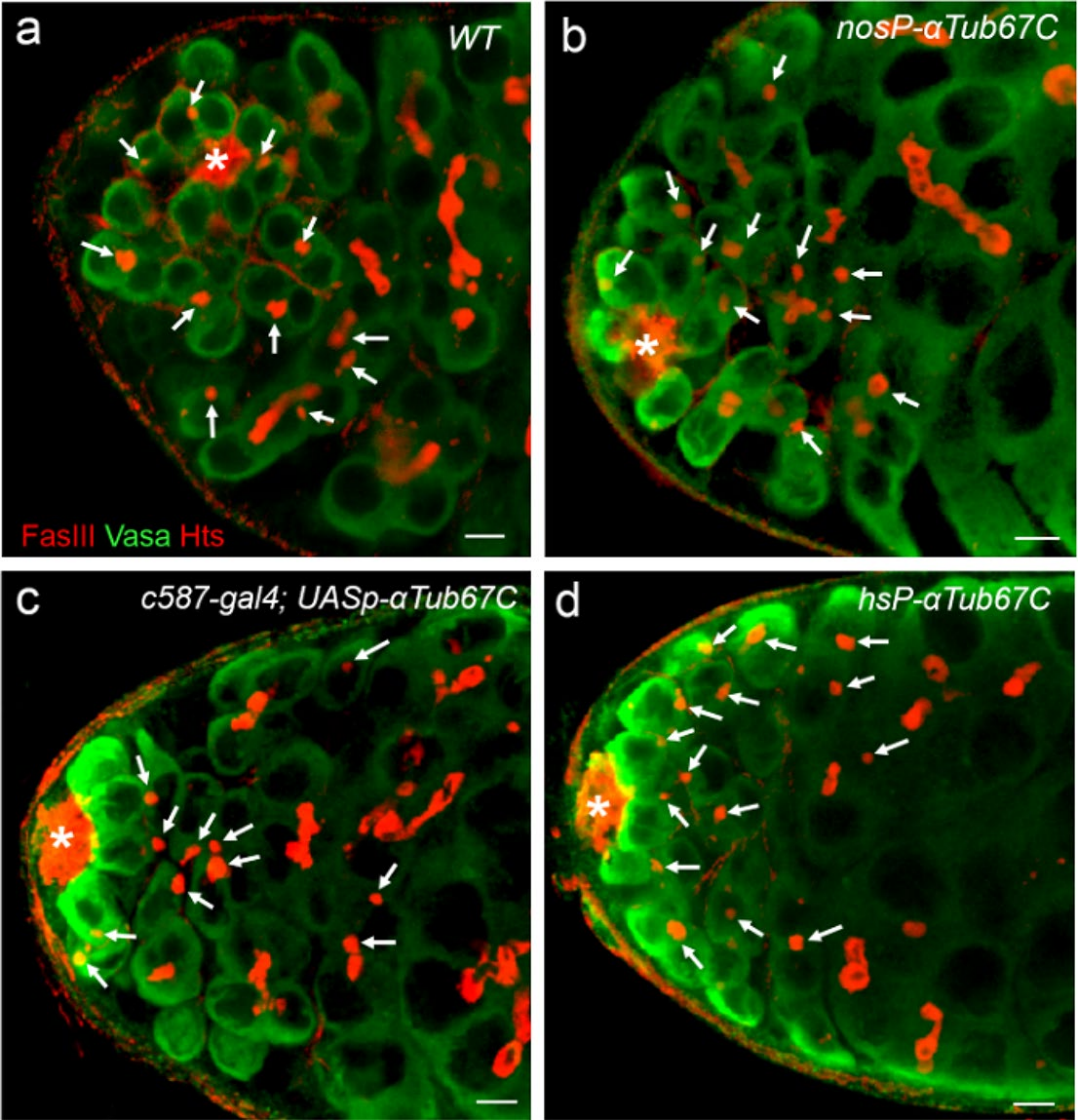
Overexpression of *αTub67C* fails to dramatically increase the number of GSC/GBs. Testes were stained with anti-Fas III antibody (red, hub with asterisk), anti-Hts antibody (red, fusomes), and anti-Vasa antibody (green, germ cells). Testes were collected from wild-type (**a**), P{*nosP-αTub67C*} (**b**), and *c587-gal4;UASp-αTub67C* male flies (**c**). Testes were dissected from P{*hsP-αTub67C*} male flies (**d**), which were cultured at 37 ℃ for 1.0 h three times per day. Spectrosomes-containing GSCs and GBs are indicated by arrows. *Oregon-R* was used as the wild-type flies. Scale bars: 5 μm.

Given the fact that deficiency of *αTub67C* resulted in loss of male GSCs, meantime, no enhanced apoptosis rates were found in *αTub67C* mutant testis GSCs, we hypothesized that ectopic overexpression of α4-Tubulin (*αTub67C-*encoding protein) might promote GSCs proliferation or/and delay GBs differentiation. To test this hypothesis, we stained the testes with anti-fas III, anti-Hts and anti-Vasa antibodies to visualize hub cells, fusomes and germ cells, respectively. Both GSCs and GBs can be identified by anti-Vasa antibody staining, and meantime by carrying spherical fusomes (spectrosomes) (Fig. 1a), and GBs undergo four times of successive cell division and generates a 16-cell germline cyst, interconnected by a branched fusome that can be visualized by anti-Hts antibody (Fig. 1a,b). According to the method described previously^19^, we measured the numbers of spectrosome-containing GSCs and GBs (SGAG) in testes from wild-type (*Oregon*) and *αTub67C*-overexpression flies, at day 5 after eclosion. We found that, in wild-type, the average number of SGAG was 11.6 per testis (n = 61) (Fig. 5a). By contrast, the numbers of SGAG from two *αTub67C*-overexpression alleles, *nosP-αTub67C* and *c587-gal4*; *UASp-αTub67C*, were 11.7 (n = 66) and 11.6 (n = 70) per testis, respectively (Table 3 and Fig. 5b,c). These results demonstrated that, compared to wild-type, there was no apparent increase in GSC/GBs number.

**Table 3:** The analyses of the average number of germ cells carrying spectrosomes in *αTub67C*-overexpressing testes. SD, standard deviation. n, Number of examined testes. ^∗^*P* > 0.05, ^#^*P* < 0.05 unpaired *t*-test, compared to *Wild-type* at day 5 post-eclosion.

Genotype	The average number of germ cells carrying spectrosomes in fly testis with *αTub67C-*overexpressing (Mean ± SD)
*Oregon*	11.6 ± 1.3 (n = 61)
*nos*P-*αTub67C*	11.7 ± 1.4 (n = 66)*
c587-gal4;UASp-*αTub67C*	11.6 ± 1.5 (n = 70)*
*hs*P-*αTub67C* (37 ℃)	13.1 ± 1.5 (n = 62)^#^

To confirm these results, we generated a new transgenic line of P{*hsP-αTub67C*}, in which the *αTub67C* cDNA was positioned downstream of the *hs70* promoter. We overexpressed *αTub67C* in testes by heat-shock, at 37 °C, for 60 min each time, for a total of three times a day. After five consecutive days of heat-shock treatments, we counted the average numbers of SGAG. We found that, compared to wild-type flies, the number of SGAG was slightly increased to an average of 13.1 SGAG per testis (n = 62) (Table 3 and Fig. 5d). Taken together, these data suggest that an enhanced α4-Tubulin activity is not sufficient to promote GSCs proliferation or block GBs differentiation.

## Discussion

Previous studies have reported that the mutation in *αTub67C* gene has an involvement of α4-Tubulin in multiple cellular processes such as spindle maintenance and elongation, sperm aster formation, the development of central and peripheral nervous system, centrosome positioning and progression of the cleavage division^24–28,44^. Here, we have revealed a novel function of the *αTub67C* gene in GSCs maintenance in *Drosophila* testis. The *αTub67C* gene encodes the α4-Tubulin protein in fruit fly, besides α4-Tubulin, there are three other α-Tubulins, α1-Tubulin (encoded by *αTub84B*), α2-Tubulin (encoded by *αTub85E*) and α3-Tubulin (encoded by *αTub84D*)^45^. After finding that *αTub67C* was required for GSCs maintenance in male flies, we subsequently performed a small scale of RNAi-mediated screen assay in other three α-Tubulins-coding genes (*αTub84B, αTub84D* and *αTub85E*) to determine whether these three α-Tubulins were likely involved in maintaining male GSCs. According to the methods described previously^39^, we specifically knocked down three α-Tubulins-coding genes (*αTub84B, αTub84D* and *αTub85E*) in fly testes by combining P{*UASp-shRNA-αTubulin*} with P{*nosP-gal4*}. We did not observe the GSCs loss phenotype (Supplementary Table S3). The results probably indicate that different α-Tubulin share different function, and the lack of *αTub67C* can’t be substituted by another *α-tubulin* gene. It is reasonable for the different member of a gene family plays a different role.

Since the *αTub67C* mutation doesn’t affect the GSCs adhesion to the hub cells in cell level (Fig. S2), whether the *αTub67C* gene regulates the expression level of E-cadherin? To address the issue, we performed reverse-transcription (RT) and performed qPCR experiments to measure the E-cadherin mRNA level with the *rp49* gene as a reference. The data show that there is no difference in the expression level of E-cadherin between the wild-type and *αTub67c* mutant testes (*P* > *0.05*) **(**Fig. S5a**)**. Given that JAK-STAT signaling pathway in CySCs is required for the maintaining GSCs^9,10^, we also conducted RT-qPCR to detect the Stat mRNA level in fly testes. Similarly, contrast to the wild-type, there is no apparent increase level in Stat expression (*P* > *0.05*) **(**Fig. S5b**)**. The above results suggest that these two genes (*E-cadherin* and *Stat*) are not transcriptionally controlled by the *αTub67c* gene.

Given that both intrinsic and extrinsic deficiency of *αTub67C* resulted in loss of male GSCs, we propose that the lost GSCs possibly undergo premature differentiation or go to the apoptosis-mediated cell death. Therefore, we examined the apoptosis in GSCs, and found no enhanced apoptosis rates in the *αTub67C* mutants. We guess that *αTub67C* mutation probably induce pre-differentiated GSCs. If so, overexpression of *αTub67C* maybe repress GSC/GBs differentiation, and increase the number of GSC/GB cells. However, we did not observe the increased numbers of GSC/GBs in *αTub67C*-overexpressed testes, suggesting that the ectopic αTub67C-overexpression has no effects on promoting GSCs self-renewal or suppressing GBs differentiation. Whether *αTub67C* affects the GBs’ differentiation? To address the issue, we analyzed the number of GBs and 2-, 4-, 8-, 16-cell spermatogonia between the wild-type and *αTub67c* mutant testes. We found that there was no difference in the average number of GBs and 2-, 4-, 8-, 16-cell spermatogonia between the wild-type and *αTub67C*^*3*^ mutant testes at day 7 after eclosion (*P* > 0.05) (Supplementary Table S4). The results indicate that *αTub67C* fails to control the GB’s differentiation into spermatocytes.

Both Dpp and Gbb, the two ligands from somatic cells, are essential for the maintenance of male GSCs in the *Drosophila*, and function as local signals in niche cells in fly testis^6^. Meantime, the Dpp/Gbb signaling activities are restricted to GSCs and GBs^6,51,52^. Interestingly, the gene *bam* is not expressed in either kind of cell, which triggered us to detect the *bam* expression pattern using *bam-GFP* transgenic reporter. The results show that the mutation in *αTub67C* fail to change the expression pattern of *bam* in GSC/GBs in *Drosophila* testes. These observations indicate that *αTub67C* functions downstream of or parallel to *bam*, and is independent of Gbb/Dpp-*bam* signaling pathway.

It is well known that the heterodimers composed of α/β-Tubulin is the major structural constituent of microtubules, the roles of which include mechanical strength, intracellular trafficking and chromosome segregation^24–28^. The α-Tubulin protein plays extensive roles by forming the microtubule (a polymeric structure). In addition, α-Tubulin also independently functions by the monomeric form. As an example, monomeric α-Tubulin fosters c-Jun protein stability by protein–protein interaction, and is required for c-Jun’s translocation and activity^53^. But for a given cellular event, which form of α-Tubulins (polymeric or monomeric) is involved in it? In this paper, the deficiency of α4-Tubulin protein encoded by *αTub67C* possibly influence the microtubule formation, and finally leads to the GSCs loss phenotype. To test the possibility, we analyzed the ER distribution in germ cells between the wild-type and *αTub67c* mutant testes. We stained testes from 14-day-old flies with ER-Tracker probe (a small molecule-conjugated with fluorescent dye to specifically label ER) to indirectly reflect the distribution of microtubules (supplementary Fig. S6). We observed that, compared to the wild-type, the ER distribution in germ cells (GSCs, GBs and spermatogonia) from *αTub67C* mutants was arranged uniformly and disorderly (Fig. S6a’,b’). The results indicate that the disorganized distribution of microtubules in *αTub67C* mutants results in the GSCs loss phenotype, and further experiments are needed to verify this hypothesis in the future.

## Conclusion

This study characterizes the *αTub67C* gene, encoding α4-Tubulin protein, which plays an essential role in the regulation of GSCs’ fate in *Drosophila* testis by using genetic strategies. The phenotypic assay of *αTub67C* mutants and FLP/*FRT*-mediated mitotic recombination analyses show that *αTub67C* is required both intrinsically and extrinsically for male GSC maintenance. *αTub67C* is not required for Dpp/Gbb signaling silencing of *bam* expression, suggesting that it functions in a bam-independent manner.

## Materials and methods

### *Drosophila* stocks

All fly stocks were raised at 25 ℃ on a standard fly medium, except those with special requirements. *Oregon-R* was used as a wild-type strain. The following strains were obtained from Bloomington Stock Center: *αTub67C*^*3*^ (#2245, X-ray), *αTub67C*^*RX2.1*^ (#43,950, X-ray), *αTub67C*^*1*^ (#1750, EMS), *FRT79D/TM*_*3*_ (#2024) and *hs-FLP*; *Ubi-GFP*, *FRT79D/TM*_*3*_ (#5825) alleles. The following lines were also used for experimentation: *c587-gal4*, *nosP-gal4* and *bamP-GFP*^19,34^. The line *UASp-shRNA-αTub67C* (#24,297) is got from Vienna *Drosophila* Resource Center. The *attP*-containing strains (#25,709 and #25,710) from Bloomington Stock Center were used as the host for phiC31-mediated transformation^31^.

### Plasmid constructs

The *pattB*-*UASp*, *pattB*-*nosP* and *pattB*-*hsP* vectors (abbreviated as *UASp*, *nosP* and *hsP*) were constructed according to a previous method^39^. To make the *UASp-αTub67C*, *hsP-αTub67C* and *nosP-αTub67C* constructs, total RNA was isolated from wild-type testes and reverse-transcription was performed, using the methods described previously^19^. Then the total cDNA was used as a template in PCR reactions to amplify the *αTub67*-coding sequence (P1/P2 as primers, Table S5), which was subcloned to *UASp*, *nosP* and *hsP*, with *AscI* and *NotI.* To generate the *attB*-*αTub67C-gDNA* construct, the genomic DNA (gDNA) was prepared from wild-type flies, as described previously^19^, which was used as template to amplify the 7.3 kb length of the *αTub67C* gDNA fragment (P3/P4 as primers, Table S5). Then, this fragment was subcloned to *nosP* with the restriction enzymes, *SbfI* and *NotI*.

### Immunohistochemistry and imaging

Testes were prepared for immunohistochemistry, as described previously^19^. Primary antibodies were used: rabbit anti-Vasa (1:500, Santa Cruz), rabbit anti-GFP (1:500, Invitrogen), mouse monoclonal anti-Fasciclin III and anti-Hts antibody (1:100, DSHB). The following secondary antibodies were used at a 1:1000 dilution: goat anti-rabbit Alexa 488 and goat anti-mouse Alexa 555 (Molecular Probe, Abcam), DAPI (dye, Yeasen) and ER-Tracker (Molecular Probe, Beyotime) were used to visualize cellular nuclei and ER, respectively. All samples were examined using a Leica fluorescent microscope, and micrographs were taken using an Olympus confocal FV1000 microscope.

### Quantitative real-time PCR (qPCR)

Total RNA was extracted from wild-type and mutant fly testes by using Trizol reagent (Sangon), then cDNA was transcribed, according to the manufacturer’s protocol (Takara). Quantitative PCR was run on a CFX96 Touch ((BioRad) to measure total *αTub67C* mRNAs with *rp49* as reference, according to the manufacturer’s protocol (SYBR Premix EX Taq™ II qPCR Kit, Takara). The following primers were used in this assay (Table S6).

### Generation and analysis of GSC clones

The FLP/*FRT*-mediated mitotic recombination technique was used to generate mutant GSCs, GBs and spermatogonia clones, as described previously^19^. For example, to generate *αTub67C*^*3*^ mutant GSCs clones, males of *hs-FLP*; *Ubi-GFP,FRT79D/ αTub67C*^*3*^*,FRT79D* and genotypes *(hs-FLP*;*FRT79D, Ubi-GFP/FRT79D* as the wild-type control) were produced by standard genetic crosses. 2-day-old adult males were heat-shocked for 90 min at 37 °C, three times per day. After 4 consecutive days of heat-shock treatment, testes were dissected for antibody staining at days 2, 7, 14 after the last heat-shock treatment. GSC clones were identified by a lack of GFP expression, as well as from their attachment position to the hub cells. GBs and spermatogonia clones were identified by GFP-negative staining, as well as rely on being far away from niche cells.

### Apoptotic cell detection

Apoptotic cell analyses were carried out using the terminal deoxynucleotidyl transferase-mediated dUTP nick end-labeling (TUNEL) technique. The GSCs from wild-type and *αTub67C* mutant testes were incubated in the reagent (1:20 dilution of the terminal deoxynucleotidyl transferase solution), then in label solution (nucleotide mixture) for 1 h at 37 °C. Fixation and Cy3-dU detection were described previously^39^.

### Statistical analysis

A Chi-square test, or Student’s t-tests were used to assess relationships between allelic variables. The level of statistical significance was set at *P* < 0.05.

## Supplementary Information

Supplementary Information.
