# Supplementary material for: α-Tubulin Regulates the Fate of Germline Stem Cells in Drosophila Testis
Source: Sci Rep. 2021 May 20;11:10644. doi: 10.1038/s41598-021-90116-7 (PMC8138004; doi:10.1038/s41598-021-90116-7)
Supplement: Supplementary file 1 — Supplementary Information. [file 41598_2021_90116_MOESM1_ESM.docx]

α-Tubulin Regulates the Fate of Germline Stem Cells in *Drosophila* Testis

Xiaoqian Tao^1^, Yunqiao Dou^1,2^, Guangyu Huang^1,2^, Mingzhong Sun^2^, Shan Lu^2^, Dongsheng Chen^1,2,3,^*

**^1^**Anhui Provincial Key Laboratory of the Conservation and Exploitation of Biological Resources, College of Life Sciences, Anhui Normal University, Wuhu 241000, China.

**^2^**Anhui Provincial Key Laboratory of Molecular Enzymology and Mechanism of Major Diseases, **^3^**College of Life Sciences, Anhui Normal University, Wuhu 241000, China.

**^3^**The Institute of Bioinformatics, College of Life Sciences, Anhui Normal University, Wuhu 241000, China

*****Correspondence: [cds2001@ahnu.edu.cn](mailto:cds2001@ahnu.edu.cn).

| **Genotype** | **The average number of GSCs in fly testis at different ages (Mean ± SD)** | |
| --- | --- | --- |
|  | **Day 1** | **Day 14** |
| *αTub67C^3^/αTub67C^1^* | 6.1±0.8 (n=62) | 3.8±1.0 (n=67) |
| P{*attB*-*αTub67C-gDNA*}; *αTub67C^3^/αTub67C^1^* | 7.8±0.7 (n=69) | 7.6±1.1 (n=63) * |
| *αTub67C^1^/αTub67C^RX2.1^* | 6.6±0.9 (n=70) | 4.3±1.0 (n=69) |
| P{*attB*-*αTub67C-gDNA*}; *αTub67C^1^/αTub67C^RX2.1^* | 7.9±1.0 (n=65) | 7.8±1.2 (n=67) * |
| *αTub67C^3^/αTub67C^RX2.1^* | 6.1±1.0 (n=71) | 4.4±0.9 (n=64) |
| P{*attB*-*αTub67C-gDNA*}; *αTub67C^3^/αTub67C^RX2.1^* | 7.7±0.9 (n=66) | 7.9±1.0 (n=67) * |

**Table S1.** The rescue assay of *αTub67C* mutants by the transgenic line of P{*attB*-*αTub67C-gDNA*}. SD, standard deviation. n, Number of testes examined. ^∗^𝑃 < 0.01, unpaired *t*-test, compared with the corresponding *αTub67C* mutant at day 14.

| **Genotype** | **The percentages of GFP negative-marked GSCs in *Drosophila* testis at different ages** | | |
| --- | --- | --- | --- |
|  | **Day 2** | **Day 7** | **Day 14** |
| *Ftr79D control* | 66.4% (n=68) | 52.7% (n=71) | 44.3% (n=61) |
| *αTub67C[RX2.1],Frt79D* | 52.4% (n=63) | 23.0% (n=77) | 3.7% (n=65) |
| *αTub67C[3],Frt79D* | 59.9% (n=62) | 19.8% (n=72) | 6.1% (n=73) |
| *αTub67C[1],Frt79D* | 63.3% (n=70) | 20.7% (n=80) | 7.5% (n=63) |
| *NosP-Tub67C ;αTub67C[RX2.1],Frt79D* | 56.1% (n=70) | 45.2% (n=67) | 49.0% (n=77) |
| *NosP-αTub67C; αTub67C[3],Frt79D* | 57.6% (n=63) | 43.8% (n=62) | 47.5% (n=67) |
| *NosP-αTub67C; αTub67C[1],Frt79D* | 56.7% (n=67) | 48.6% (n=67) | 51.3% (n=69) |

**Table S2.** The analyses of the percentages of negatively GFP-marked GSC clones in FRT control and αTub67C mutant alleles at day 2, 7 and 14. n, the total number of GSCs examined.

| **Genotype** | **The average number of GSCs in *Drosophila* testis at different ages (Mean ± SD)** | | |
| --- | --- | --- | --- |
|  | **Day 1** | **Day 7** | **Day 14** |
| *Oregon-R* | 8.1±1.0 (n=77) | 7.6±0.9 (n=80) | 7.3±1.0 (n=89) |
| *UASp-shRNA-αTub84B; nosP-gal4* | 8.2±0.9 (n=78) | 7.2±0.7 (n=86) | 7.0±0.9 (n=88)^∗^ |
| *UASp-shRNA-αTub84D; nosP-gal4* | 8.0±1.2 (n=78) | 7.0±1.0 (n=70) | 6.8±1.0 (n=90)^∗^ |
| *UASp-shRNA-αTub85E; nosP-gal4* | 7.9±1.3 (n=89) | 7.8±1.2 (n=65) | 6.9±1.0 (n=71)^∗^ |

**Table S3.** Phenotypic assay for the *αTub84B-, αTub84D-, αTub85E*-specific knockdown in *Drosophila* testis. All the examined flies were cultured at 29℃. SD, standard deviation. n, Number of testes examined. ∗*𝑃 >0.05*, unpaired t-test, compared with the wild-type control at day 14.

| **Genotype** | **The average numbers of GB and 2-, 4-, 8-, 16-cell spermatogonia**  **in *Drosophila* testis (Mean ± SD)** | | | | | |
| --- | --- | --- | --- | --- | --- | --- |
|  | **GB cells** | **2-cell spermatogonia** | **4-cell spermatogonia** | **8-cell spermatogonia** | **16-cell spermatogonia** | **Total number of testis** |
| *Oregon-R* | 11.1±1.4 | 9.1±1.2 | 6.7±1.1 | 4.7±1.1 | 4.8±1.0 | n=12 |
| *αTub67C^3^/ αTub67C^3^* | 8.7±1.1^∗^ | 8.5±1.4^∗^ | 5.9±1.1^∗^ | 4.9±1.0^∗^ | 4.7±1.2^∗^ | n=11 |

**Table S4.** The analyzed the number of GB and 2-, 4-, 8-, 16-cell spermatogonia between the wild-type and *αTub67C^3^* mutant testes. n, Number of testes examined. ∗*𝑃 >0.05*, unpaired t-test, compared with the wild-type (*Oregon*) control at day 7 after eclosion.

| **No.** | **Primer Sequences** | **Restriction enzymes (underlined)** |
| --- | --- | --- |
| P1 | 5’-AAAGGCGCGCCATGCGCGAAGTAGTCTCCATCC-’(forward) | *AscI* |
| P2 | 5’-TTTGCGGCCGCTTAGAACTCATCGAAGTCCTCGTC-3’(reverse) | *NotI* |
| P3 | 5’-TTTCCTGCAGGGGAATCCAGTTGACTGGGTAAATTC-3’(forward) | *SbfI* |
| P4 | 5’-TTTGCGGCCGCACAGAAAACTAATGCAAGAATG-3’ (reverse) | *NotI* |

**Table S5.** Primers used for generating *αTub67C* transgenic vectors.

| **Primer names** | **Sequences of Primers** |
| --- | --- |
| *αTub67C* | 5’-TGCCTATGCCCCACTGATGT-3’ (forward) |
|  | 5’-CCACATCACCCCTGTAAAGC-3’ (reverse) |
| *E-cadherin* | 5’-CCATTGACAACAGTGGCTGC-3’ (forward) |
|  | 5’-CGTGACGGTGACCTCTTTCA-3’ (reverse) |
| *stat* | 5’-AACCAATTCTGTAAGGAGCC-3’ (forward) |
|  | 5’-GTTGATGAAGCCCATAATGC-3’ (reverse) |
| *rp49* | 5’-CACTTCATCCGCCACCAGTC-3’ (forward) |
|  | 5’-CGCTTGTTCGATCCGTAACC-3’ (reverse) |

**Table S6.** The primers in doing qPCR assay.


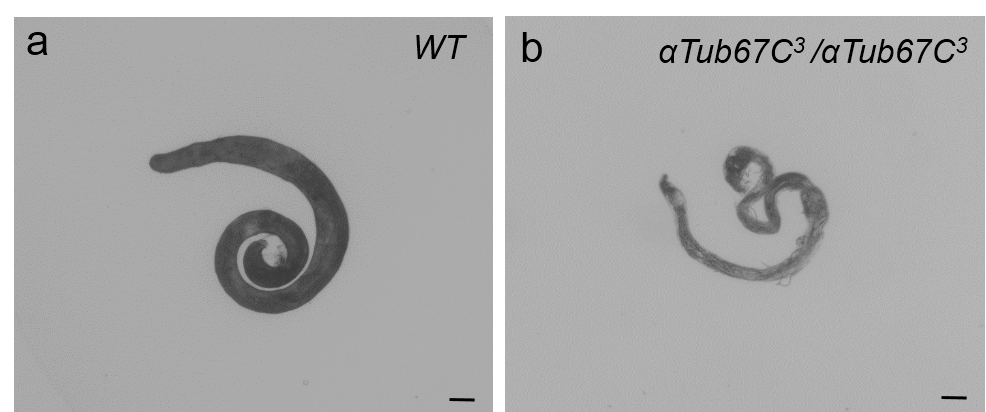


**Fig. S1** *αTub67C^3^* homozygous mutant flies exhibited shrunk testes at day 10 after eclosion. (a) Testes from wild-type. (b) *αTub67C^3^* mutants. The testes of *αTub67C^3^* mutants became more thinner and smaller than the *wild-type* . Scale bars: 100μm.

**
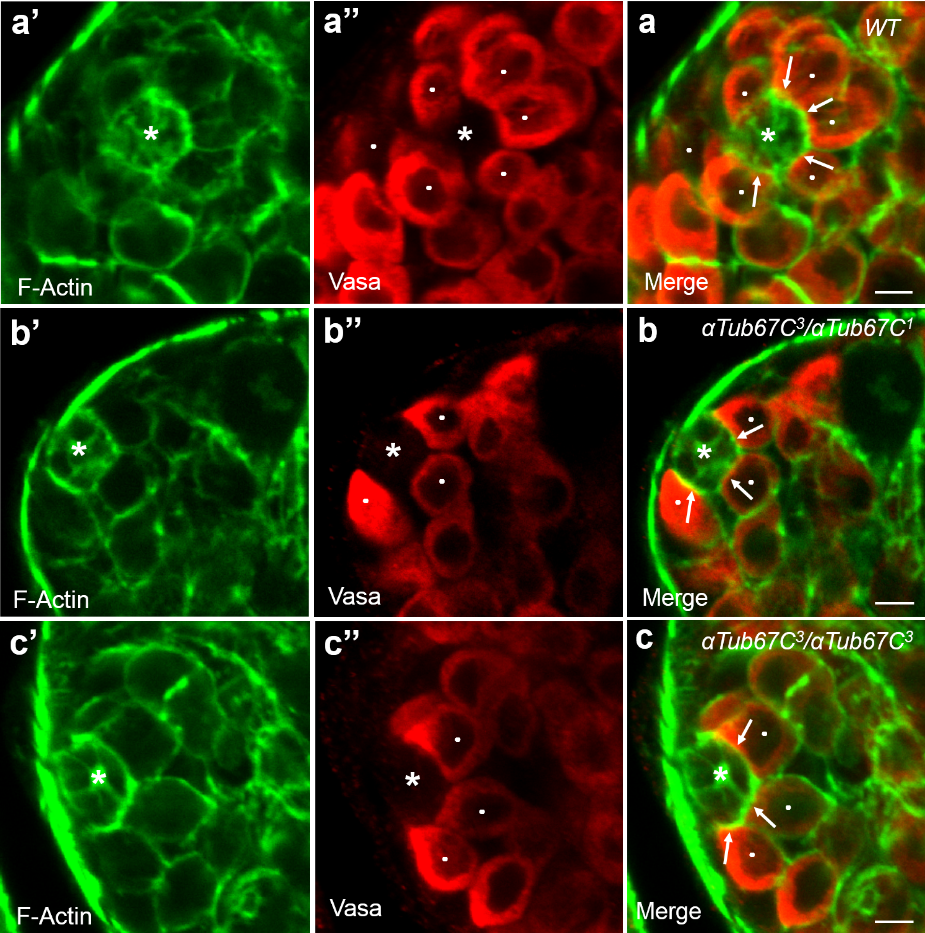
**

**Figure S2. *αTub67C* fails to affect cell-cell (GSC and hub cell) adhesion.** Testis from wild-type (a’-a), *αTub67C^3^/αTub67C^1^* mutant (b’-b) and *αTub67C^3^/αTub67C^3^* mutant fly (c’-c) collected at day 14 after eclosion. Testes were stained with FITC-conjugated Phalloidin (green, hub cells and germ cells) and anti-Vasa antibody (red, germ cells). The hub was indicated by asterisk (green), GSCs were noted by white dots (red). The boundaries of GSCs and hub cells were indicated by arrows. There was no difference in cell-cell adhesions (GSC and hub cell) between *αTub67C* mutants and wild-type testes. Scale bars: 5μm.


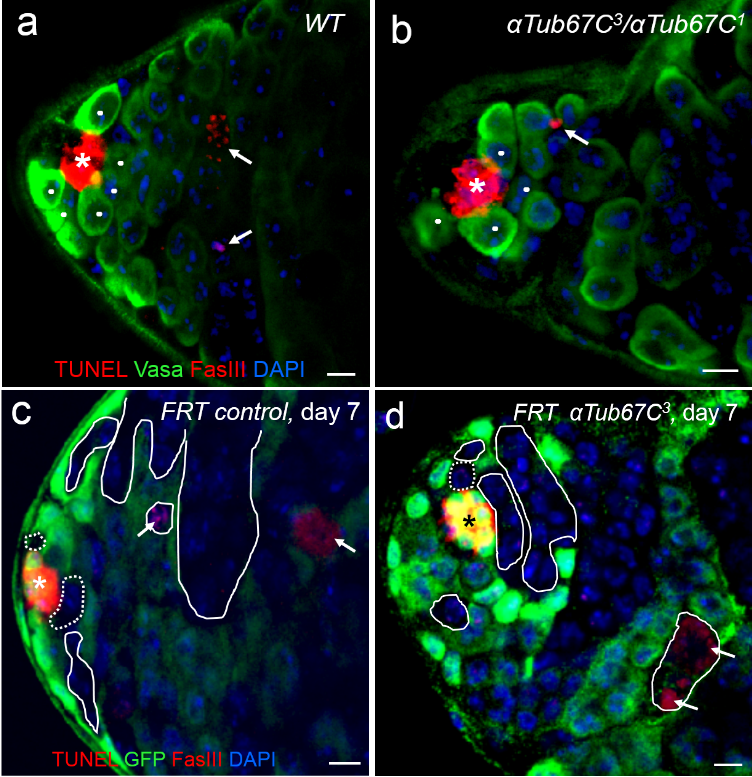


**Fig. S3** The mutations in *αTub67C* gene fails to lead to apoptosis in GSCs. (a) Testes from wild-type. (b) *αTub67C^3^/αTub67C^1^* mutant testis. (a-d) Testes were labeled by TUNEL (red, indicated by arrows) and stained with anti-FasIII (red) and DAPI (blue). Testes were stained with anti-Vasa antibody (green) (a-b) and anti-GFP antibody (green) (c-d). (c) Testes from wild-type of *FRT* control. (d) *αTub67C^3^* mutant GSC clones. Hubs were indicated by asterisks. GSCs were noted by white dots (a-b) and GSC mutant clones were noted by broken lines (c-d). GB/spermatogonia clones were indicated by circles. Scale bars: 5μm.


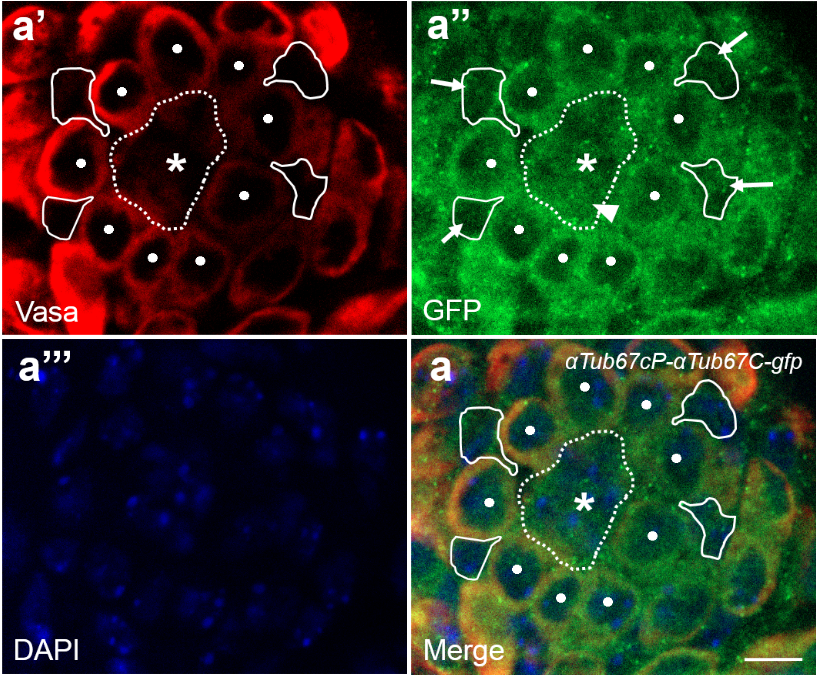


**Figure S4. The gene αTub67C is expressed ubiquitously in adult fly testis.** Testis bearing a transgene P{*αTub67CP-αTub67C-GFP*} was stained with anti-Vasa antibody (red) to label the GSCs and germ cells (a’), anti-GFP antibody (green) to show the αTub67C expression (a’’), and DAPI dye (blue) to visualize the nuclei (a’’’). The αTub67C-GFP fusion protein were expressed ubiqitously in all cell types including somatic cells (e.g. hub noted by broken line and asterisk, CySCs marked by circle) and germline cells (e.g. GSCs noted by white dot), suggesting the αTub67C is expressed in CySCs (circles, arrows), in hub (broken line, arrowhead) and in GBs. Scale bars: 5μm.


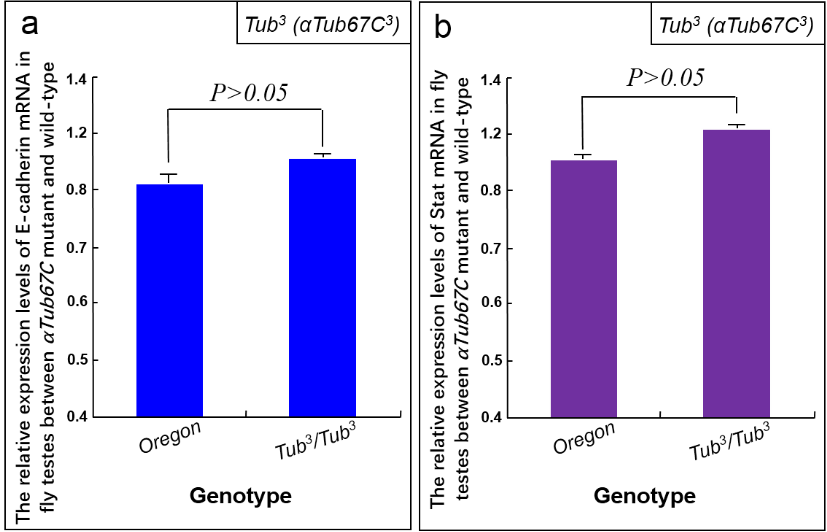


**Figure S5. Quantitative PCR analyses of the gene mRNA levels.** QPCR analyses of E-cadherin mRNA levels (a) and Stat mRNA levels (b) in testes between wild-type (*Oregon*) and *αTub67C* mutants (*αTub67C^3^/αTub67C^3^*).


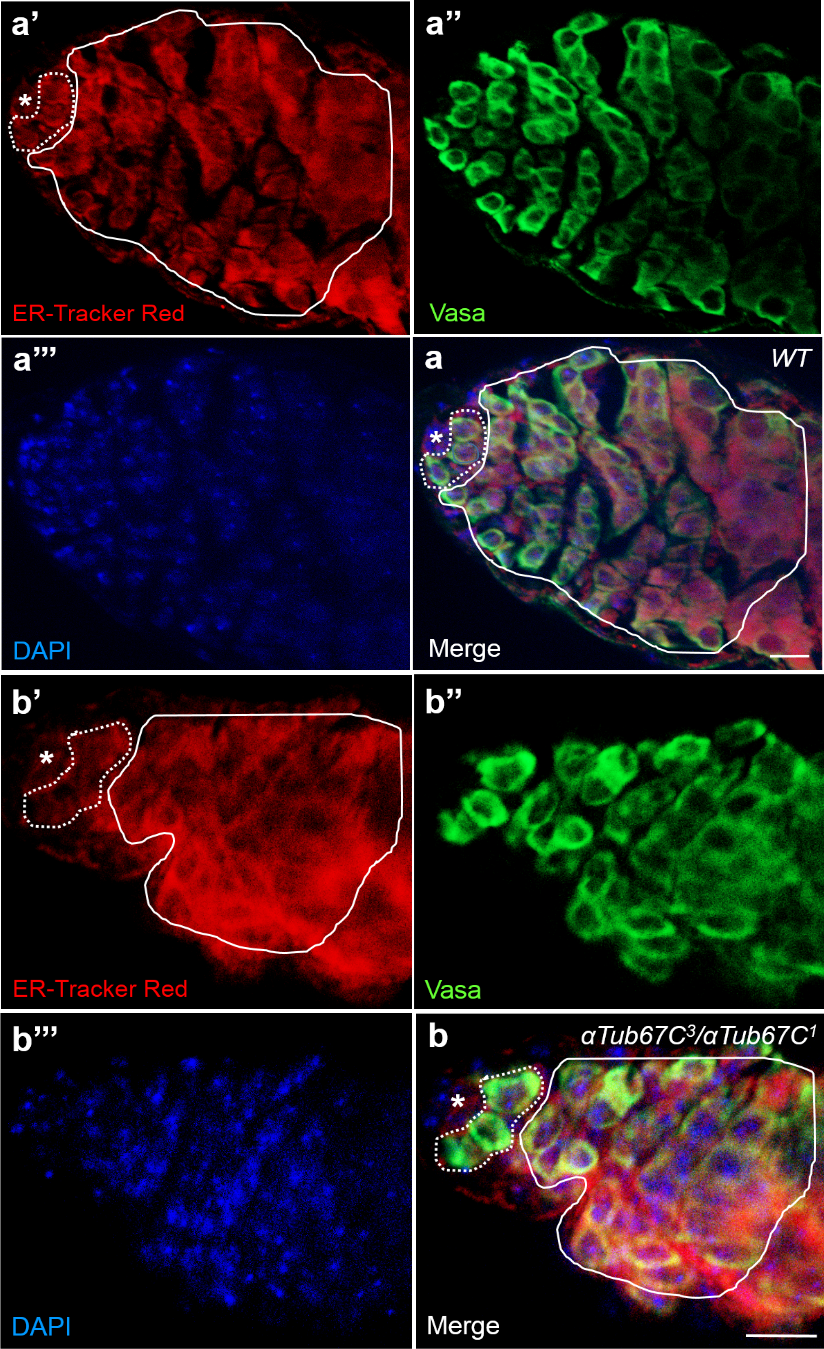


**Figure S6. The distribution of ER in testicular cells is affected by *αTub67C* in *Drosophila*.** Testes from wild-type (a’-a) and *αTub67C^3^/αTub67C^1^* mutants (b’-b) were labeled by ER-Tracker (red) and stained with anti-Vasa (green) and DAPI (blue) at day 14 after eclosion. The hub was noted with asterisk, GSCs noted with broken line, GBs and spermatogonia cells marked with circle. Compared to the wild-type, the ER distribution in GSCs and other germ cells (GBs and spermatogonia) in *αTub67C* mutants was disorderly distributed. Scale bars: 10μm.
